# Generative artificial intelligence–assisted visual art therapy improves cognition, anxiety, and depression in community-dwelling older adults

**DOI:** 10.3389/fpubh.2026.1874071

**Published:** 2026-08-07

**Authors:** Shi Zhuo, Min Wang, Yi Zhuo

**Affiliations:** 1 School of Art and Design, Hubei University of Technology, Wuhan, China; 2 Minda Hospital of Hubei Minzu University, Enshi, China

**Keywords:** community-dwelling older adults, generative artificial intelligence, mild cognitive impairment, Montreal cognitive assessment, visual art therapy

## Abstract

**Objective:**

To examine whether generative artificial intelligence–assisted visual art therapy improves cognitive function and alleviates anxiety and depression in community-dwelling older adults with mild cognitive impairment.

**Methods:**

A quasi-experimental, cluster-gated design was employed in Shengli Street Community, Enshi City (September 2025–April 2026). Eighty eligible older adults were enrolled and assigned by administrative grid to an intervention group (*n* = 40) or a control group (*n* = 40). The control group continued routine community activities; the intervention group additionally received a 12-week generative artificial intelligence–assisted visual art therapy program. The primary outcome was change in Montreal Cognitive Assessment (MoCA) total score; the Hospital Anxiety and Depression Scale (HADS) served as the secondary outcome. Assessments occurred at baseline, 1 week post-intervention, and at three-month follow-up. Cohen’s d effect sizes are reported.

**Results:**

Seventy-two participants completed the study. At post-intervention, the intervention group scored significantly higher on the MoCA (d = 1.36, *p* < 0.001) and lower on HADS-Anxiety and HADS-Depression (d = 0.73–0.76, both *p* < 0.01) relative to controls. Gains were largely maintained at 3 months (MoCA d = 1.08). Fidelity reached 93.5%; no serious adverse events occurred.

**Conclusion:**

An AI-enabled, four-phase visual art therapy program can improve cognition and reduce anxiety and depression in community-dwelling older adults with mild cognitive impairment, with effects partially sustained at 3 months. The observed benefits reflect the synergistic effects of the multi-component intervention as a whole, rather than the isolated contribution of any single element.

## 1 Introduction

China has entered a phase of deep population aging, and mental health has become central to healthy aging. Yet community-based psychological services for older adults remain constrained by workforce shortages, limited service formats, and low levels of digital adoption (1). Mild cognitive impairment, a transitional state between normal aging and dementia, affects roughly 15 to 20 percent of the global population aged 60 and above (2). It frequently co-occurs with anxiety and depressive symptoms, and together cognitive decline and emotional distress can reinforce one another, accelerating functional deterioration (3). Scalable, low-barrier, sustainable community interventions are therefore urgently needed.

Art therapy—grounded in nonverbal expression and creative engagement—is well suited to older adults whose language and cognitive capacities are in decline, and meta-analytic evidence confirms small-to-moderate benefits for global cognition and depressive symptoms in this population (4). Traditional art therapy, however, depends heavily on trained therapists and presupposes artistic skills that many older adults do not possess, which limits its scalability (5).

Recent advances in generative artificial intelligence, particularly diffusion-based text-to-image models, offer a way forward: by converting natural-language descriptions into high-quality images almost instantaneously, they shift the core demand of art making from manual dexterity to verbal narration and aesthetic judgment, substantially lowering the barrier to participation (6–8). Despite this promise, empirical studies that systematically combine generative artificial intelligence with art therapy and deliver the combined intervention to community-dwelling older adults are absent from the literature. No theoretical account of the interaction mechanisms has been advanced, few protocols have been tailored to the cultural and cognitive profiles of Chinese elders, and the existing evidence base lacks well-designed trials reporting effect sizes, fidelity metrics, and follow-up data.

We hypothesize that a generative AI art therapy that transforms manual dexterity into oral narration and aesthetic judgment will selectively enhance language functions (naming, discourse coherence) and executive functions (inhibitory control, cognitive flexibility) in older adults by increasing the demands on semantic retrieval, episode construction, and aesthetic evaluation, and that its effects will be significantly superior to those of regular community activities, which provide only general social stimulation.

The theoretical basis for this hypothesis is as follows. Oral narration involves retrieving concepts from semantic memory and constructing syntactic expressions online, a process that relies heavily on the left frontotemporal language network, including the posterior superior temporal gyrus (Wernicke’s area) and the inferior frontal gyrus (Broca’s area). Aesthetic judgment requires evaluating the degree of match between a generated image and internal aesthetic standards and making value-based decisions among multiple candidate images, which demands sustained monitoring and revision by the dorsolateral prefrontal cortex and thus strongly activates the executive control network. This intervention therefore carries inherent cognitive specificity: it does not “activate the brain” in a diffuse manner but precisely targets the language production pathway and the executive evaluation pathway. This constitutes the core logic underlying our expectation that its effects will surpass those of conventional art therapy (which relies on manual dexterity and visuomotor coordination). The present study does not directly compare these two modalities; rather, it tests whether this AI-enabled psychosocial intervention as a whole yields measurable cognitive and emotional improvements relative to an active control condition matched for social contact and time investment.

We therefore designed and tested a four-phase generative artificial intelligence–assisted visual art therapy protocol in the Shengli Street Community, Enshi City—a mid-sized, inland community with modest service infrastructure characteristic of China’s less developed interior. We hypothesized that, relative to routine community activities, the intervention would yield greater improvements in the Montreal Cognitive Assessment total score (H1) and greater reductions in Hospital Anxiety and Depression Scale anxiety and depression subscale scores (H2), and that these between-group differences would remain detectable at a three-month follow-up (H3).

## 2 Conceptual foundations

### 2.1 Operational definition

We term the intervention *generative artificial intelligence–assisted visual art therapy*. Operationally, it is a digitally mediated, nonpharmacological intervention in which older adults, guided by a trained group facilitator, engage in a three-step cycle: (a) human–AI collaborative creation, wherein the older adult supplies narrative content and aesthetic judgment while the generative artificial intelligence executes visual translation; (b) sharing of the visual products within the group; and (c) facilitated group feedback. The older adult functions as the director of the creative process, the generative artificial intelligence as the brush, and the facilitator as the process catalyst (9).

### 2.2 Theoretical framework

Four theoretical perspectives underpin the intervention logic model. Cognitive reserve theory posits that novel, multimodal cognitive activities—such as those demanded by generative artificial intelligence–facilitated creation—can build compensatory neural networks and strengthen cognitive resilience (10). Expressive arts therapy theory holds that artistic creation and symbolic expression bypass linguistic defenses and access emotional material directly (11). Human–AI collaboration theory delineates a functional boundary we adopt: the human retains control over semantic content and aesthetic decisions; the AI supplies generation and variation (12). Social support theory accounts for the informational, emotional, and belongingness support mobilized during group sharing (13).

## 3 Methods

### 3.1 Design

**Figure 1:**
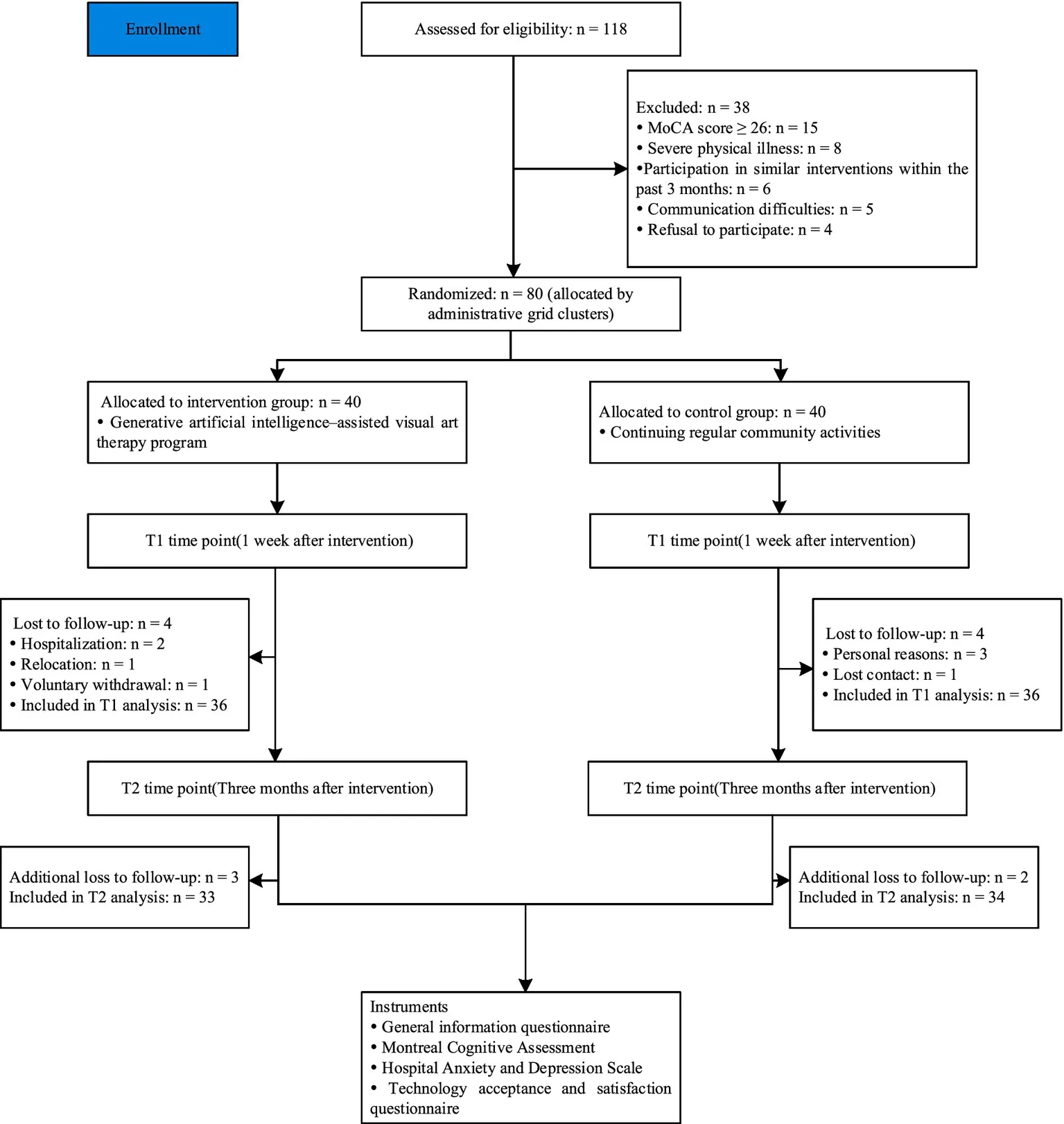
The overall process figure.

We employed a quasi-experimental, cluster-gated, repeated-measures design with assessments at baseline (T0), 1 week after the intervention (T1), and at a three-month follow-up (T2). The overall process is shown in Figure 1.

### 3.2 Sample size

An *a priori* power calculation was performed in G*Power 3.1. Drawing on the effect size reported by Masika and colleagues (14) for MoCA change following visual art therapy in mild cognitive impairment (Cohen’s d = 0.65), and setting *α* = 0.05 (two-tailed), 1 − *β* = 0.80, and an allocation ratio of 1:1 for an independent-samples t-test, the minimum required sample was 37 per group. Anticipating approximately 15 percent attrition, we planned to recruit 44 participants per arm. Constrained by the realities of community-based recruitment, we enrolled 40 per group.

### 3.3 Participants

Participants were drawn by convenience sampling from the older adult residents of the Shengli Street Community. The community is partitioned into six administrative grids; to minimize contamination across conditions, we assigned Grids 1 through 3 to the intervention arm and Grids 4 through 6 to the control arm.

Inclusion criteria were as follows: (a) aged 60 years or older; (b) stable residence in the community for at least 6 months; (c) a MoCA-Beijing Version score below 26, indicating mild cognitive impairment or elevated risk of cognitive decline; (d) corrected visual acuity of at least 0.3 (pocket vision screener) and the ability to hear speech at normal conversational volume at 1 meter, plus successful completion of a prescreening tablet operation task (power on, open app, tap option, swipe screen); and (e) provision of written informed consent.

Exclusion criteria were as follows: (a) a confirmed diagnosis of Alzheimer’s disease or another dementia, with a Clinical Dementia Rating of 1 or above; (b) severe psychiatric illness (for example, schizophrenia, bipolar disorder) or debilitating physical conditions precluding participation—specifically, New York Heart Association functional class III or higher, chronic obstructive pulmonary disease requiring long-term oxygen therapy, or stroke or myocardial infarction within the preceding 6 months; (c) participation in a similar cognitive or psychological intervention within the past 3 months; and (d) severe communication difficulties that would render scale administration unreliable.

Over the course of the trial, attrition occurred as follows: in the intervention arm, two participants withdrew because of hospitalization, one relocated out of the community, and one voluntarily discontinued; in the control arm, three withdrew for personal reasons and one was lost to follow-up. Thus, 72 participants completed both T0 and T1 assessments. At T2, 67 participants provided data (intervention, *n* = 33; control, *n* = 34).

### 3.4 Intervention

#### 3.4.1 Control condition

The control group continued with the community’s standard activities: a monthly health education lecture on chronic disease management and seasonal wellness, daily access to an activity center where older adults could freely play mahjong, Chinese chess, and read newspapers and books, and organized square dancing and park fitness activities. The research team did not modify any of these activities; attendance frequency was simply recorded.

We selected “regular community activities” (chorus, Chinese chess and card games, reading clubs) as the active control based on the following considerations. This control condition is highly matched to the intervention group in terms of frequency of social contact, novelty of activities, and weekly time commitment, thus effectively controlling for the nonspecific effects of group companionship and structured activities. Critically, these regular community activities contain no generative language production or aesthetic decision-making component—participants either conform to predetermined rules (Chinese chess and card games) or consume content passively (reading clubs), without the need to actively construct narratives or perform aesthetic evaluations. This control design therefore maximally isolates the target cognitive mechanisms that the present intervention is intended to engage. Furthermore, we did not set a conventional art therapy (e.g., painting, collage) as a control condition, not out of oversight, but because the two rely on fundamentally distinct cognitive pathways—conventional art therapy centers on manual dexterity and visuomotor coordination, whereas the present intervention centers on oral expression and aesthetic decision-making. A direct comparison between the two lies beyond the immediate purpose of the current study; we have added a note to this effect in Section 5.4, “Limitations,” and recommend that future research add a conventional art therapy arm for direct comparison.

#### 3.4.2 Intervention condition

Participants in the intervention arm received, in addition to the standard programming described above, a 12-week generative artificial intelligence–assisted visual art therapy program.

1. Interdisciplinary Team. The team comprised a chief physician in geriatric medicine (responsible for medical oversight and risk management), an associate professor specializing in art therapy (intervention design and supervision), two community public-health nurses (recruitment and health monitoring), and two graduate students in social work (intervention delivery and data collection). Prior to the trial, the art therapy faculty member conducted 16 h of centralized training covering geriatric psychology, operation of the generative artificial intelligence tool (a domestically compliant text-to-image model), group facilitation skills, and empathic communication. Competence was verified through standardized scenario-based assessments.
2. Intervention Protocol. Drawing on Hogan’s art therapy framework (15), cognitive reserve theory, and emotion regulation models, we constructed a four-phase program comprising Reminiscence, Reframing, Expression, and Elevation (Table 1). The intervention consisted of 12 weekly sessions, each lasting approximately 70 mins, delivered in the digital activity room of the community center in groups of six to eight.
3. Generative Artificial Intelligence System Specification. The generative artificial intelligence system used in this study was the Doubao 1.8 platform, utilizing its built-in text-to-image model, Seedream 4.0. This model is a diffusion-based text-to-image model capable of generating high-quality images in diverse styles from natural-language prompts. Throughout the study, the standard user interface of Doubao 1.8 was used for all operations, and no API was called—that is, all image generation was performed through the platform’s native interactive interface, without any back-end programmatic invocation. The considerations for selecting this platform included the following: (a) the platform has completed compliance registration in mainland China, meeting the relevant domestic data security and content management regulations; (b) the platform interface is clean and user-friendly, suitable for operation by trained facilitators assisting older adult participants in a community setting; (c) the Seedream 4.0 model has demonstrated stable performance in Chinese semantic understanding and image generation quality, responding well to the diverse types of prompts involved in this study—nostalgic scenes, emotional metaphors, and future-oriented visions alike.
4. Prompt strategy. Participants used the Doubao app’s voice input function to describe, in natural language, the image they wished to generate. The built-in speech recognition module transcribed the spoken description into text in real time, and this text was used directly as the generation prompt; we added no preset system prompts or prompt chains. For example, when a participant said, “Draw a cat basking in the sun in an old courtyard, with China rose flowers beside it,” the system used this text as the prompt for Seedream 4.0. The app itself incorporates built-in safety content filtering and default stylization parameters, making additional prompt engineering unnecessary. See Appendix A for a typical example.
5. Hyperparameters. All image generation parameters were the default settings of Doubao version 1.8; the researchers had neither the authority nor the need to modify them. According to the app’s technical documentation and our own measurements, the output resolution is 1,024 × 1,024 pixels, generation takes approximately 5–10 s, and the format is JPEG. These default settings ensure that other researchers using the same app version can replicate the interaction.

**Table 1:** The four-phase generative artificial intelligence–assisted visual art therapy protocol.

Phase (Weeks)	Theme	Core therapeutic goal	Session flow and generative artificial intelligence collaboration mechanism
Reminiscence: Memory Awakening (1–3)	*Images of Time*	Activate episodic and autobiographical memory; build group safety	① Warm-up (10 min): Soft music; display nostalgia-inducing images of old Enshi (streets, the Qing River bridge) pregenerated by the generative artificial intelligence to elicit spontaneous recollection. ② Collaborative creation (30 min): Participants describe “the person, place, or event I miss most.” Keywords (for example, “Tujia stilted house,” “the bicycle from my youth”) are entered into the generative artificial intelligence; the older adult selects the image that best matches the memory. ③ Sharing and discussion (20 min): Story-sharing around the “old photo”; the facilitator guides peers to offer positive feedback and emotional resonance.
Reframing: Cognitive Reappraisal (4–6)	*Mood Mosaic*	Externalize emotion; train visuospatial and executive functions	① Warm-up (10 min): Display generative artificial intelligence–generated abstract faces with distinct expressions alongside color cards; conduct emotion-matching games. ② Collaborative creation (30 min): Participants describe their mood using the metaphor “color + shape + weather” (for example, “a gray fog with a sliver of blue”). The generative artificial intelligence translates this into mood-tone cards and visual metaphors. Participants cut, collage, and rework the output into a mood collage. ③ Sharing and discussion (20 min): “5W1H” questioning guides artwork interpretation; participants practice identifying and accepting complex emotions.
Expression: Self-Narrative (7–9)	*Tree of Life*	Integrate life narrative; enhance self-continuity and sense of control	① Warm-up (10 min): The generative artificial intelligence generates a dynamically growing tree, symbolizing the life course. ② Collaborative creation (30 min): Using the “Tree of Life” as a scaffold, participants supply key life events (marriage, childbirth, relocation, recovery from illness) as prompts. The generative artificial intelligence cogenerates a series of narrative images, arranged into a personal life scroll. ③ Sharing and discussion (20 min): Coherent telling of one’s life story; the facilitator helps identify resilience and strengths embedded in the narrative.
Elevation: Vision Reconstruction (10–12)	*Tomorrow’s Hope*	Foster positive future orientation; promote social bonding and closure	① Warm-up (10 min): The generative artificial intelligence generates healing, future-oriented scenes (a rainbow after rain, a community garden). ② Collaborative creation (30 min): Participants imagine “something I most want to do” or “someone to whom I want to send a blessing,” and cocreate future postcards. The generative artificial intelligence inpainting function allows the addition of personal symbols. ③ Sharing and farewell (20 min): Exchange postcards within the group; share gains and future plans; compile a group digital commemorative album; complete group closure.

The technical workflow was as follows. The older adult described a memory, emotion, or imagined scene in natural language; this description served as the prompt. A trained operator entered the prompt into the generative artificial intelligence model, which produced four to eight images in varied styles within seconds. The older adult then selected, edited, and combined images according to personal preference, constructing a personalized visual work. Across the entire workflow, the older adult retained full authority over aesthetic decisions and narrative direction.

1. Ethical and Safety Procedures. All generative artificial intelligence–generated content was prescreened by the operator to ensure a positive and healthy orientation. At the start of each session, we explicitly reinforced the message that the older adult is the director and the generative artificial intelligence is merely the brush, in order to forestall any sense of techno-intimidation. Emotional responses were monitored throughout; participants who showed marked distress received one-on-one support from the therapist after the session. Adverse events (for example, technology-related anxiety, emotional crises) were managed according to a prespecified protocol and documented.
2. Treatment Fidelity. Fidelity was assessed using a self-developed checklist comprising 20 items: 12 on procedural adherence, 4 on time management, and 4 on generative artificial intelligence operation norms; each item was scored 0 to 2, for a maximum of 40 points. Two independent raters scored audio-video recordings of every session. Interrater reliability was estimated via the intraclass correlation coefficient. The overall fidelity rate was calculated as (mean actual score/theoretical maximum) × 100 percent.

### 3.5 Instruments

General information questionnaire. This self-designed instrument captured demographics (age, sex, years of education, marital status, monthly income), number of chronic conditions, and prior experience with smart devices.

Montreal Cognitive Assessment (MoCA), Beijing Version. Used for mild cognitive impairment screening and global cognitive assessment. It spans seven cognitive domains—visuospatial/executive function (5 points), naming (3), attention (6), language (3), abstraction (2), delayed recall (5), and orientation (6)—with a total of 30 points. One point is added for those with 12 or fewer years of education. The MoCA total score served as the primary outcome. The Chinese version has a Cronbach’s *α* of 0.85 (16).

Hospital Anxiety and Depression Scale (HADS). Fourteen items across two subscales—HADS-Anxiety (7 items) and HADS-Depression (7 items)—each scored from 0 to 3. Subscale totals of 0 to 7 are classified as nonclinical, 8 to 10 as borderline, and 11 or above as a probable clinical case (17). The Chinese version’s total scale Cronbach’s *α* is 0.879 (18).

Technology acceptance and satisfaction questionnaire. A brief 10-item self-report measure covering perceived usefulness (4 items), perceived ease of use (3 items), and overall satisfaction (3 items), rated on a five-point Likert scale (1 = strongly disagree; 5 = strongly agree). Cronbach’s *α* was 0.87 in our sample. Only the intervention group completed this instrument, at T1.

Assessor blinding. All MoCA assessments and HADS administrations were conducted independently by a systematically trained research assistant who was completely unaware of participants’ group assignments. This assistant was not involved in any aspect of intervention delivery and was explicitly prohibited from inquiring about or discussing participants’ group status prior to each assessment. Data entry was completed by a separate graduate student who was also blind to group assignment. This single-blind procedure ensured assessor independence.

### 3.6 Statistical analysis

Analyses were performed in SPSS 25.0, with *α* set at 0.05 (two-tailed). Normality of continuous variables was checked with the Shapiro–Wilk test. Baseline between-group comparability was assessed using independent-samples t-tests for continuous variables (reporting exact degrees of freedom) and χ^2^ tests for categorical variables (reporting degrees of freedom and sample size; Fisher’s exact test was used when expected cell frequencies were below 5).

Primary Analysis: Linear Mixed-Effects Models. The primary analytic framework for all outcome variables was the linear mixed-effects model (LMM), implemented via the MIXED procedure in SPSS 25.0 with restricted maximum likelihood (REML) estimation. The model specification was as follows: fixed effects included Group (intervention vs. control), Time (T0, T1, T2), and the Group × Time interaction; random effects included a random intercept for Participant ID to account for within-individual correlations across repeated measures. The covariance structure was selected as unstructured (UN) based on the Akaike Information Criterion (AIC). Significance testing employed Satterthwaite approximation for degrees of freedom. The primary parameter of interest was the F-test for the Group × Time interaction; a significant interaction was followed by Bonferroni-corrected simple-effects analyses to determine the specific time points at which between-group differences were statistically significant. Effect sizes (Cohen’s d) were calculated based on the model-estimated marginal means and pooled standard deviations, with 95% confidence intervals; benchmarks: 0.20 = small, 0.50 = medium, 0.80 = large (19).

Sensitivity Analyses. Two sensitivity analyses were conducted. First, intention-to-treat (ITT) analyses were performed using the last observation carried forward (LOCF) method for participants lost to follow-up. Second, to account for potential clustering effects arising from the group-based nature of the intervention (participants nested within intervention subgroups of six to eight), we fitted an additional LMM with “Intervention Subgroup” as a second-level random intercept. The intraclass correlation coefficient (ICC) was computed to quantify the magnitude of clustering effects.

MoCA Subdomain Analyses. For *post hoc* comparisons across the seven MoCA subdomains, we applied a Bonferroni correction (adjusted *α* = 0.05/7 ≈ 0.007). All *p*-values for subdomain comparisons are reported as both uncorrected and Bonferroni-corrected.

Reporting Standards. In accordance with the Frontiers statistical reporting guidelines, all t-tests are reported with exact degrees of freedom in the format t(df) = X. XX, *p* = 0. XXX; all χ^2^ tests are reported in the format χ^2^(df, N = XXX) = X. XX, *p* = 0. XXX. Exact *p*-values are reported unless *p* < 0.001. All Cohen’s d values are reported with 95% confidence intervals.

### 3.7 Ethical approval, privacy protection, and data confidentiality

#### 3.7.1 Ethical approval

This study received approval from the Ethics Committee of the Research Ethics and Science and Technology Security Committee of Hubei University of Technology (ethics approval number: [HBUT20260035]). The study was conducted in strict accordance with the ethical principles of the Declaration of Helsinki.

#### 3.7.2 AI data usage disclosure in informed consent

During the informed consent process, the researcher orally explained the following to each participant and, where applicable, their family members: (1) participants’ oral descriptions would be transcribed into text prompts and entered into an online AI image generation platform (Doubao 1.8/Seedream 4.0) to produce visual artworks; (2) prompts should not include directly identifiable personal information such as real names, identification numbers, or home addresses; (3) because a commercial platform was used, the research team could not technically guarantee that input data would not be used by the platform provider for service improvement purposes.

#### 3.7.3 Data anonymization and de-identification

All scale data (MoCA, HADS, general information questionnaire, technology acceptance questionnaire) were coded immediately upon collection, with research identification numbers replacing participants’ real names. The correspondence table linking names to research identification numbers was stored separately in encrypted form, accessible only to the principal investigator and the data manager.

#### 3.7.4 Data storage and access control

Paper-based scale data were stored in a locked filing cabinet at the School of Art and Design, Hubei University of Technology. Electronic data (including digitized scale entries, AI-generated images, and audio-video recordings for fidelity assessment) were stored on a dedicated encrypted hard drive, accessible only to authorized researchers. All electronic files were password-protected.

#### 3.7.5 Team member confidentiality obligations

All team members (including graduate student assistants) signed written data confidentiality agreements, pledging not to use any participant information obtained during the study for purposes beyond the research.

#### 3.7.6 Privacy handling of audio-video recordings

The audio and video recordings used for fidelity assessment captured only group activity processes (and did not record personally identifiable information). They were stored on an encrypted hard drive and will be destroyed within 2 years after journal publication, upon completion of data analysis. Raters signed independent agreements regarding the confidential handling of recordings.

#### 3.7.7 Data retention and destruction plan

In accordance with the research data management policy of the host institution, research data (anonymized) will be retained for a minimum of 5 years from the date of final publication for possible verification analyses. Digital copies of AI-generated images will be retained alongside the research data after anonymization. All personally identifiable data (such as original informed consent forms and the name-to-ID correspondence table) will be securely destroyed (paper documents by shredding, electronic files by secure erasure) at the end of the retention period.

## 4 Results

### 4.1 Baseline comparability

Seventy-two older adults completed both T0 and T1 assessments, with a mean age of 68.51 ± 5.82 years. The two groups did not differ significantly on any demographic variable, clinical characteristic, or baseline scale score (all *p* > 0.05). Details appear in Table 2.

**Table 2:** Baseline demographic and clinical characteristics.

Variable	Intervention (*n* = 36)	Control (*n* = 36)	Statistic	df	*p*
Age (years)	68.17 ± 5.94	68.86 ± 5.73	*t* = 0.506	70	0.615
Female, *n* (%)	22 (61.1)	19 (52.8)	χ^2^ = 0.510	1^a^	0.475
Education (years)	9.11 ± 3.42	8.97 ± 3.65	*t* = 0.166	70	0.868
Chronic conditions, *n*	1.78 ± 0.99	1.86 ± 1.05	*t* = −0.348	70	0.729
Prior smart device use, *n* (%)	27 (75.0)	25 (69.4)	χ^2^ = 0.277	1^a^	0.599
MoCA total (T0)	21.31 ± 2.98	21.44 ± 2.85	*t* = −0.203	70	0.840
HADS-Anxiety (T0)	9.67 ± 3.12	9.42 ± 3.05	*t* = 0.344	70	0.732
HADS-Depression (T0)	9.08 ± 3.45	8.89 ± 3.28	*t* = 0.245	70	0.807

MoCA, Montreal Cognitive Assessment; HADS, Hospital Anxiety and Depression Scale. Continuous variables are presented as mean ± SD and were compared using independent-samples t-tests (two-tailed); categorical variables are presented as frequency (percentage) and were compared using χ^2^ tests. For all continuous-variable independent-samples t-tests, df = 70 (n₁ + n₂ − 2 = 36 + 36–2). For all χ^2^ tests, df = 1.

a *N* = 72 for all χ^2^ tests.

As shown in Figure 1, this study employed cluster allocation rather than individual randomization. The intervention group received a 12-week (once weekly, 70 min per session), four-stage generative AI–empowered visual art therapy (platform: Doubao 1.8/Seedream 4.0). The control group continued their regular community activities (health lectures, chess and card games, square dancing, etc.). The primary outcome was the change in Montreal Cognitive Assessment (MoCA) total score from T0 to T1 and T2; secondary outcomes were the anxiety and depression subscales of the Hospital Anxiety and Depression Scale (HADS). Assessments were conducted using a single-blind design, with assessors blinded to group assignment.

### 4.2 Treatment fidelity

Across the 12 sessions, the mean fidelity score was 37.4 ± 1.6 out of 40, yielding an overall protocol adherence rate of 93.5 percent (95% CI, 91.2–95.8). The interrater intraclass correlation coefficient was 0.89 (95% CI, 0.84–0.93), indicating good consistency between raters. In short, the intervention was delivered largely as designed.

### 4.3 Primary outcome: MoCA

The LMM analysis revealed a significant Group × Time interaction effect on MoCA total scores, *F*(2, 103.47) = 39.82, *p* < 0.001. Simple-effects analyses with Bonferroni correction showed no significant between-group difference at T0 (estimated marginal mean difference = −0.13, 95% CI: −1.52, 1.26, *p* = 0.840). At T1, the intervention group scored significantly higher than the control group (estimated marginal mean difference = 4.61, 95% CI: 3.05, 6.17, t(103.25) = 5.91, *p* < 0.001, d = 1.38). At T2, the between-group difference remained significant (estimated marginal mean difference = 3.79, 95% CI: 2.12, 5.46, t(101.84) = 4.52, *p* < 0.001, d = 1.09; Table 3).

**Table 3:** MoCA total scores across time points (mean ± SD).

Time point	Intervention	Control	*t*	*df*	*p*	Cohen’s *d* (95% CI)
T0	21.31 ± 2.98	21.44 ± 2.85	−0.203	70	0.840	−0.05 (−0.51, 0.42)
T1	25.89 ± 2.43**	22.06 ± 3.15	5.774	70	**< 0.001**	1.36 (0.85, 1.87)
T2	25.12 ± 2.67**	21.83 ± 3.28	4.378	65	**< 0.001**	1.08 (0.58, 1.58)

MoCA, Montreal Cognitive Assessment. T0 = baseline; T1 = one week post-intervention; T2 = three-month follow-up. Between-group comparisons at each time point were conducted using independent-samples t-tests (two-tailed), with exact df, t-values, *p*-values, and Cohen’s d with 95% CI reported. The primary analysis employed linear mixed-effects models (LMM); the t-tests at individual time points are reported for descriptive completeness and should be interpreted in the context of the significant Group × Time interaction from the LMM, F(2, 103.47) = 39.82, *p* < 0.001. Effect size benchmarks: 0.20 = small, 0.50 = medium, 0.80 = large. **indicates statistical significance at the *p* < 0.01 level. Bold text serves to emphasize.

The ITT sensitivity analysis (LOCF) yielded results consistent with the per-protocol analysis: Group × Time interaction, *F*(2, 108.52) = 36.17, *p* < 0.001.

At T1, the intervention group had gained 4.58 points on the MoCA relative to baseline (95% CI, 3.72–5.44), whereas the control group had gained only 0.62 points (95% CI, −0.06 to 1.30). The between-group effect size was large (d = 1.36). At the three-month follow-up, the intervention group’s MoCA scores had declined slightly—by 0.77 points from T1—but remained well above baseline (net gain, 3.81 points) and still significantly higher than those of controls (d = 1.08; *p* < 0.001). The intention-to-treat sensitivity analysis yielded results consistent with the per-protocol analysis. ᵃ At T2: intervention n = 33, control n = 34; therefore df = 33 + 34–2 = 65. At T0 and T1: intervention n = 36, control n = 36; df = 70.

Table 4 presents the pre-to-post change in each of the seven MoCA subdomains, with between-group comparisons using independent-samples t-tests (all df = 70) and Bonferroni correction. After correction, robust between-group differences favoring the intervention remained for visuospatial/executive function (t(70) = 4.315, *p* < 0.001, corrected *p* = 0.001, d = 0.97), attention (t(70) = 3.385, *p* = 0.001, corrected *p* = 0.007, d = 0.79), and delayed recall (t(70) = 5.626, *p* < 0.001, corrected *p* < 0.001, d = 1.33). The differences in naming (t(70) = 2.571, uncorrected *p* = 0.012, d = 0.59), language (t(70) = 2.988, uncorrected *p* = 0.004, d = 0.69), and abstraction (t(70) = 2.988, uncorrected *p* = 0.004, d = 0.69) were no longer significant after correction. Orientation improved modestly in both groups, with no significant between-group difference (t(70) = −1.572, *p* = 0.121, d = −0.37).

**Table 4:** Pre-to-post change in MoCA subdomains (T1 − T0; mean ± SD).

Cognitive domain	Intervention Δ(*n* = 36)	Control *Δ*(*n* = 36)	*t*	*p* (Uncorrected)	*p* (Bonferroni)	*d (95% CI)*
Visuospatial/executive	0.64 ± 0.72	−0.04 ± 0.60	4.315	< 0.001	0.001*	0.97 (0.48, 1.46)
Naming	0.25 ± 0.44	0.03 ± 0.29	2.571	0.012	0.084	0.59 (0.12, 1.06)
Attention	0.72 ± 0.97	0.06 ± 0.67	3.385	0.001	0.007*	0.79 (0.31, 1.27)
Language	0.33 ± 0.48	0.03 ± 0.38	2.988	0.004	0.028	0.69 (0.22, 1.16)
Abstraction	0.33 ± 0.48	0.03 ± 0.38	2.988	0.004	0.028	0.69 (0.22, 1.16)
Delayed recall	0.81 ± 0.82	−0.25 ± 0.77	5.626	< 0.001	< 0.001*	1.33 (0.82, 1.84)
Orientation	0.50 ± 0.70	0.78 ± 0.80	−1.572	0.121	0.847	−0.37 (−0.83, 0.09)

MoCA, Montreal Cognitive Assessment. Δ = T1 − T0 (positive values indicate improvement). Between-group comparisons were conducted using independent-samples t-tests (two-tailed). All t-tests have df = 70 (n₁ + n₂ − 2 = 36 + 36–2). The Bonferroni correction was applied to control for multiple comparisons across the seven subdomains: adjusted significance threshold α = 0.05/7 ≈ 0.007. Cohen’s d and 95% CI are reported for each subdomain comparison.*Statistically significant after Bonferroni correction (*p* ≤ 0.007). Bold text serves to emphasize.

### 4.4 Secondary outcome: HADS

The LMM analysis revealed a significant Group × Time interaction effect on HADS-Anxiety, *F*(2, 102.61) = 7.48, *p* < 0.001, and on HADS-Depression, *F*(2, 103.18) = 8.93, *p* < 0.001. Simple-effects analyses confirmed that both HADS-Anxiety and HADS-Depression scores in the intervention group dropped significantly from baseline to T1 (both *p* < 0.01), while control group scores remained essentially unchanged. At T2, the intervention group showed a slight rebound from T1, but scores remained significantly below baseline and below those of controls (HADS-Anxiety, *p* = 0.013; HADS-Depression, *p* = 0.008). Effect sizes were in the medium-to-large range (Table 5).

**Table 5:** HADS scores across time points (mean ± SD).

Measure	Time	Intervention	Control	*t*	*df*	*p*	Cohen’s *d* (95% CI)
HADS-Anxiety	T0	9.67 ± 3.12	9.42 ± 3.05	0.344	70	0.732	0.08 (−0.38, 0.54)
	T1	6.81 ± 2.54**	8.97 ± 3.20	−3.181	70	**0.002**	0.73 (0.26, 1.20)
	T2	7.12 ± 2.78**	9.03 ± 3.35	−2.564	65	**0.013**	0.63 (0.14, 1.12)
HADS-Depression	T0	9.08 ± 3.45	8.89 ± 3.28	0.245	70	0.807	0.06 (−0.41, 0.52)
	T1	6.42 ± 2.44**	8.53 ± 3.11	−3.205	70	**0.002**	0.76 (0.28, 1.24)
	T2	6.78 ± 2.61**	8.67 ± 3.05	−2.716	65	**0.008**	0.69 (0.20, 1.18)

HADS, Hospital Anxiety and Depression Scale. Each subscale (HADS-Anxiety and HADS-Depression) contains 7 items, with a score range of 0–21. Between-group comparisons at each time point were conducted using independent-samples t-tests (two-tailed), with exact df, t-values, *p*-values, and Cohen’s d with 95% CI reported. The primary analysis employed linear mixed-effects models (LMM); the t-tests at individual time points are reported for descriptive completeness. Significant Group × Time interactions from the LMM: HADS-Anxiety, F(2, 102.61) = 7.48, *p* < 0.001; HADS-Depression, F(2, 103.18) = 8.93, *p* < 0.001. **p < 0.01 versus same-group T0. T2: intervention, *n* = 33; control, *n* = 34. Bold text serves to emphasize.

The effect sizes for both HADS subscales showed modest attenuation from T1 to T2: HADS-Anxiety, d = 0.73 → 0.63 (approximately 13.7% reduction); HADS-Depression, d = 0.76 → 0.69 (approximately 9.2% reduction). Both remained within the medium-to-large range, suggesting that the emotional benefits of the intervention were partially sustained at 3 months.

### 4.5 Technology acceptance and adverse events

The intervention group’s mean technology acceptance score at T1 was 4.32 ± 0.48 out of 5, with subscale scores of 4.41 ± 0.52 (perceived usefulness), 4.18 ± 0.55 (perceived ease of use), and 4.37 ± 0.49 (satisfaction). Taken together, these values suggest that participants found the generative artificial intelligence tool both usable and worthwhile. No serious adverse events occurred across the 12-week intervention. Two participants reported mild technology-related anxiety during their first exposure to the generative artificial intelligence tool (nervousness about operating the tablet, fear of pressing the wrong button); both cases resolved after one-on-one coaching and encouragement by the second session. No participant required suspension of the intervention because of emotional distress.

## 5 Discussion

### 5.1 Effects on cognitive function

The most striking finding, we think, is the magnitude of the MoCA gain in the intervention group relative to controls: d = 1.36 at T1, which is well above the pooled effect reported in the art therapy meta-analytic literature (standardized mean difference ≈ 0.48)^5^. This observation, though tentative, raises the possibility that the generative artificial intelligence component may amplify the cognitive benefits of visual art therapy through distinct pathways.

It is important to emphasize that the effect sizes reported here reflect the overall impact of the multi-component intervention package. The generative AI tool, group facilitation, reminiscence-based narrative exercises, and peer social support likely contributed synergistically to the observed cognitive gains. Based on the current single-control design, we cannot—and do not—claim causal effects for the AI component alone.

The subdomain pattern offers some clues. After correction, the most robust gains appeared in visuospatial/executive function, attention, and delayed recall—a pattern that aligns reasonably well with the cognitive demands inherent in the generative artificial intelligence–assisted workflow. To produce a satisfactory image, the participant must mentally construct a visual scene (drawing on visuospatial processing), sustain attention while comparing multiple generated images and making aesthetic judgments (calling on executive control), and then encode the selected visual output in relation to personal experience (engaging episodic memory). All of this fits comfortably within the cognitive reserve framework: novel, multimodal, cognitively engaging activities are thought to build and reinforce compensatory networks.

It is worth noting that the control group exhibited a slight decline in delayed recall (*Δ* = −0.25). This finding is consistent with the natural trajectory of memory decline in mild cognitive impairment and, we would suggest, indirectly underscores the protective role the intervention may play for this particularly vulnerable domain. Orientation, in contrast, improved in both groups. The routine social activities available in the community setting appear sufficient to maintain temporal and spatial orientation, and the generative artificial intelligence intervention did not confer additional benefit here.

### 5.2 Emotion externalization and cognitive reappraisal: a distinctive pathway

The reductions in anxiety and depression we observed (HADS-Anxiety, d = 0.73; HADS-Depression, d = 0.76) are broadly consistent with the established benefits of group art therapy (20). Yet here, too, the generative artificial intelligence component may introduce a mechanism that has received little empirical attention: what we term instantaneous emotion externalization and visualization.

During the “Mood Mosaic” sessions, older adults described their internal emotional states in metaphorical language—a gray fog, for example. Within seconds, the generative artificial intelligence rendered that fog into a concrete, viewable, modifiable visual artifact. This process, we argue, amounts to a form of externalized cognitive reappraisal. The theoretical logic is as follows: when an emotional state is represented as an external object, psychological distance increases, and with it, flexibility in emotion regulation (21). One participant’s spontaneous remark during a sharing session captures something of this dynamic: “Seeing that gray thing hanging on the wall, it does not feel so heavy inside anymore” (quoted from process notes; consent for use obtained). Though anecdotal, the statement echoes the sequence spelled out in Gross’s process model of emotion regulation (22)—deployment of attention followed by cognitive change.

Two additional factors likely contributed. The generative artificial intelligence output, being often unexpectedly beautiful, generated a collective sense of positive surprise and aesthetic pleasure that quickly lightened the group atmosphere. And the sharing of personally meaningful images provided a low-defensive anchor for conversation, easing social isolation. The “Tree of Life” and “Tomorrow’s Hope” phases, for their part, guided participants toward reconstructing a coherent, affirmative life narrative—a process that carries particular weight in countering existential distress and depression in later life.

### 5.3 Clinical and public health significance

We see significance at three levels. At the individual level, generative artificial intelligence assistance relocates the barrier to creative participation from the hand to the mouth and the mind, giving older adults with no prior art training access to flow experiences and the satisfactions of creative self-expression. At the service delivery level, a tablet-based protocol reduces training costs relative to traditional one-on-one art therapy; a single facilitator can guide a group of six to eight—a meaningful efficiency gain for resource-constrained settings. At the system level, communities in central and western China face acute shortages of psychological service resources. A digital model of this kind may help narrow the urban–rural and regional gap in geriatric mental health care, offering a practical tool in support of the “Healthy China 2030” agenda.

### 5.4 Limitations

Several limitations should be kept in view. First, and most critically, the causal attribution problem. Our study used cluster-gated, nonrandom assignment and lacked an active comparison condition—for instance, a conventional art therapy arm without generative artificial intelligence. Consequently, we cannot disentangle the effects attributable specifically to the generative artificial intelligence component from those arising from weekly group companionship and structured creative activity more generally. Furthermore, even within the AI-assisted protocol as delivered, the current design does not permit us to separate the cognitive effects of AI tool use per se (prompt construction, multi-image comparison, aesthetic decision-making) from those of the reminiscence-based intervention content (autobiographical memory retrieval, repeated retrieval practice, narrative elaboration). These two sets of mechanisms are procedurally inseparable in the present protocol, as every session integrated AI-mediated image generation with autobiographical material. The terminology we adopted throughout—generative artificial intelligence–assisted visual art therapy—is meant to describe the intervention as a whole, not to imply single-component attribution. A three-arm randomized controlled trial (usual care versus conventional art therapy versus generative artificial intelligence–assisted art therapy) is the obvious next step. To further isolate each component’s unique contribution, future studies should additionally consider a reminiscence-only arm (structured autobiographical recall without AI image generation) and a creation-only arm (AI image generation from neutral prompts without reminiscence guidance), enabling a full component-dismantling design. Second, the actual sample size (72) fell slightly short of the *a priori* target (74), which may have underpowered the secondary outcomes and subdomain analyses. Third, this was a single-site study in a mid-sized community in inland China; generalizability to large eastern cities or rural villages remains uncertain. Fourth, the MoCA, despite its robust psychometrics, is a performance-based screening instrument and cannot fully rule out practice effects or assessor expectancy. Fifth, at T2 we lost five participants (7.0 percent)—a modest attrition rate, but one that may introduce bias. Sixth, regarding data governance, this study used a commercial generative AI platform (Doubao 1.8 with Seedream 4.0) via its standard user interface without API-level data protection agreements or local deployment. Consequently, we cannot guarantee that prompts entered into the system were excluded from the platform provider’s model training or service improvement datasets. Although no directly identifiable personal information was intentionally included in prompts, and operators were instructed to generalize any inadvertently disclosed identifiers, the data governance framework of this study lacked the rigor of enterprise-grade privacy protection. Future studies should consider API-based deployment with contractual data-processing agreements or local deployment of open-source models to ensure full data sovereignty. Seventh, although the present active-control design effectively controls for the nonspecific effects of social contact, activity novelty, and participation time, it cannot rule out the potential contribution of the novelty associated with using a digital device (tablet computer) per se to the cognitive and emotional outcomes. Future studies may add a “standard tablet use” group—for example, participants use a tablet to browse neutral images or play simple digital games—in order to further isolate the specific effects of generative AI interaction and separate the novelty effect of the digital device from the specific cognitive mechanisms of AI-assisted creation.

## 6 Conclusion

**Figure 2:**
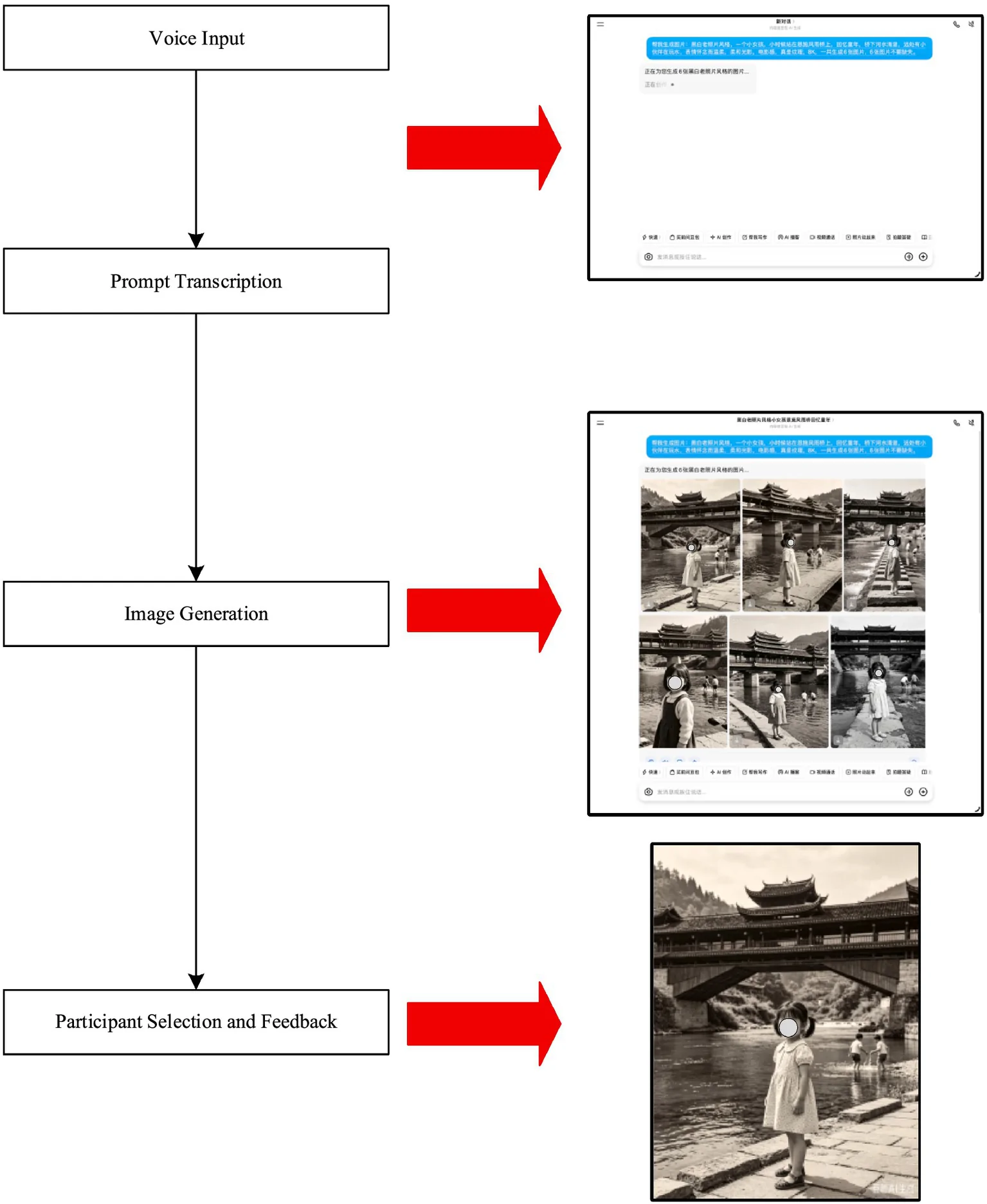
Typical AI interaction example.

Our findings suggest that a 12-week, four-phase AI-enabled visual art therapy program—a multi-component psychosocial intervention—can meaningfully improve global cognitive function and reduce symptoms of anxiety and depression among community-dwelling older adults with mild cognitive impairment, with therapeutic gains partially sustained 3 months after the intervention. By shifting the creative threshold from manual skill to verbal narration and aesthetic choice, and by enabling the externalization and cognitive reappraisal of difficult emotions, this model offers a novel digital pathway for mental health maintenance in community settings. The approach exemplifies a technology-enabled, human-centered logic that may usefully inform the development of community-based digital geriatric mental health services. We emphasize that the observed effects reflect the synergistic impact of the intervention package as a whole; the specific contribution of the AI component remains to be isolated in future component-dismantling designs. A large-scale, multisite, three-arm randomized trial incorporating objective neurophysiological indices and component-control arms is now needed to confirm these effects and clarify the underlying mechanisms (Figure 2).

## Data Availability

The raw data supporting the conclusions of this article will be made available by the authors, without undue reservation.

## Appendix A

Typical AI interaction example

As shown in Figure 2, a representative anonymized interaction sequence from the “Mood Mosaic” phase (Week 5) is provided below. The participant (female, 71 years, MoCA baseline = 22) provided additional written consent for the use of anonymized screenshots in academic publication.

Step 1: Voice Input. The participant stated in natural language: “黑白老照片风格, 一个小女孩, 小时候站在恩施风雨桥上, 回忆童年, 桥下河水清澈, 远处有小伙伴在玩水” (A little girl is standing on the Fengyu Bridge in Enshi, with clear river water flowing beneath and her friends playing with water in the distance).

Step 2: Prompt Transcription. The Doubao application’s built-in speech recognition module transcribed the oral description in real time. The transcribed text served directly as the prompt for the Seedream 4.0 model. No system prompts or prompt engineering were applied.

Step 3: Image Generation. The Seedream 4.0 model generated six images within approximately 7 s (default parameters: resolution 1,024 × 1,024 pixels, JPEG format).

Step 4: Participant Selection and Feedback. The participant browsed the six images, selected the one she felt best matched her internal emotional state, and stated during the sharing session: “Seeing that little girl, it does not feel so heavy inside anymore.”
